# Whisky-Inspired Active Matter

**DOI:** 10.1021/acsami.6c00489

**Published:** 2026-04-20

**Authors:** Khalifa Mohamed, Kelly Henze, Juliane Simmchen

**Affiliations:** † Department of Pure and Applied Chemistry, 3527 University of Strathclyde, Glasgow G1 1XL, U.K.; ‡ Department of Chemistry, 9169 TU Dresden, Dresden 01069, Germany; § IPF Dresden, Institute of Physical Chemistry and Polymer Physics, Dresden 01069, Germany

**Keywords:** microswimmers, whisky, sulfides, dimethyl sulfide, active matter, Janus particle, redox reaction

## Abstract

Whisky is a valuable commodity around the world, partly due to its rich heritage and complex taste and scent. Analyses of the final product show a complex symphony of chemicals ranging from sulfides, phenols, and tannins to esters, serving as an imprint of the manufacturing and aging processes that each whisky goes through. A well-known reactivity between copper and sulfides during whisky making inspires the determination of whether this reactivity can be harnessed on the microscale. Our system uses spherical Cu@SiO_2_ Janus particles in aqueous solutions of dimethyl sulfide to determine the capacity of DMS to serve as a chemical fuel for the propulsion of microswimmers. Other sulfur-containing molecules, e.g., aliphatic mono- and disulfides, thiophene, thiazole, and thiols, are tested as well to determine their capacity for Cu@SiO_2_ Janus particle actuation. Our investigation identifies that water-soluble aliphatic monosulfides successfully actuate the microswimmers with velocities reaching up to 30 μm·s^–1^. We propose a possible underlying reaction mechanism for these fuels and discuss alternatives, e.g., DMSO and Cu_2_O are formed when DMS is used as a chemical fuel. To further reflect the whisky-inspired type of fuel, ethanol–water mixtures as the swimming medium are examined, and their effects on the swimming behavior of Cu@SiO_2_ Janus particles are evaluated. This work expands the array of fuels that can be used to propel microswimmers.

## Graphical abstract

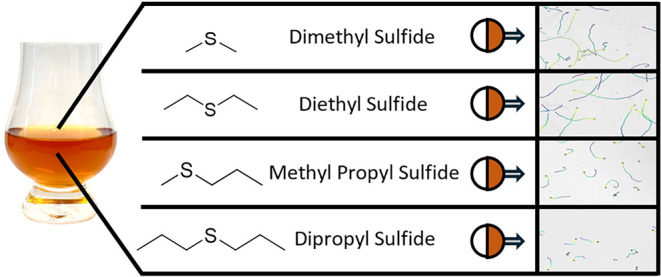

## Introduction

Scottish whisky is an internationally known product cherished by many who appreciate its complexity, suiting a wide range of tastes. It is a highly valued commodity that made up £5.4 billion of Scottish exports in 2024, equating to 44 bottles sold each second.^1^ The sensorial profile in each bottle is provided by a plethora of chemical compounds, each contributing to the overall sensory experience. Some of the very pronounced characteristics of whisky are created by sulfurous compounds.^2–6^

The overall process of whisky fabrication starts with barley, which is soaked in water to allow it to sprout, a process that releases simple sugars from complex carbohydrates. These sugars are the substrate for fermentation, which is mainly performed by yeast after mashing (Figure S1). After fermentation, the so-called wash contains about 10% of alcohol. Distilling the low alcohol fermented mixture, concentrates it into greater ethanol content.^7^ Traditionally, copper has been the material of choice for distillation stills^8^ for two main reasons: its high thermal conductivity and its chemical reactivity. This process not only increases the ethanol content through fractional distillation but also enables chemical interactions between the wash and copper. During whisky production, several techniques are employed by distilleries to influence the final composition of flavor and aroma compounds,^4^ with the distillation process in copper stills itself playing a central role. The concentration of copper in Scottish whisky varies significantly depending on the producer; however, in general, it is on the order of hundreds of ng·l^–1^.^9^ Copper is particularly known to affect the sulfurous compounds present in the liquid^4,8^ during the distillation process and later during maturation in casks in its ionic form, all of these are used to tune the spirit’s characteristics.^8,10^ For example, a reaction mechanism of ionic Cu with thiols has been shown in a wine model by Kreitman et al. to selectively remove H_2_S.^11^

Copper as a metal is in strong demand for its desirable physical and chemical properties, for a favorable electric conductivity and cost in electronics,^12–14^ as a catalyst in different chemical reactions,^15–18^ and in medicine as a potent antimicrobial agent.^19–22^ While chemically driven active matter has traditionally been propelled using catalytic reactions, mostly H_2_O_2_,^23–25^ some N_2_H_4_,^26^ and water splitting.^27,28^ New methods are continuously being developed to further the range of fuels available to power micromotors. Reactions involving copper can proceed via redox reactions, initially pioneered by Sen’s group^29^ with a nano battery concept using halogens as fuel. Galvanic exchange reactions are mostly performed with noble metal conjugate acids using a less noble half-metal Janus particle, leading to a deposition of the noble metal on the particle’s surface.^30,31^

Chemical interactions between copper and sulfides were an inspiration to recreate similar interactions on the microscale with the use of spherical Cu@SiO_2_ Janus particles in aqueous and ethanol–water solutions of various sulfur-containing molecules and observe their behavior. A somewhat related reaction mechanism in Ren et al. showed that Cu powder reacted with sulfides in methanol to produce the respective sulfoxides and sulfones of the reactant.^18^ This work and the association of ethanol in whisky were also inspirations to recreate similar interactions on the microscale with Cu@SiO_2_ spherical Janus particles in water–ethanol mixtures and observe their behavior.

## Results and Discussion

To assess the microscale effects of the reaction between copper and sulfur-compounds, one of the chemically simplest sulfides, dimethyl sulfide (DMS) in aqueous solutions, has been tested as fuel for Cu-capped Janus particles (Figures S2 and S3 for SEM images and manufacturing scheme). DMS has shown to propel Cu@SiO_2_ Janus particles (MSD vs time lag, Figure 4A) in solutions of a concentration range from 6.25 to 200 mmol·l^–1^, Figure 1A with the Cu-cap forward at all concentrations. Up to concentrations of 50 mmol·l^–1^ of DMS, the average velocity of the particles progressively increases, since with increasing fuel, more product is formed and the driving force of the motion increases due to the steeper chemical gradient that forms between the reaction reactants and products. Beyond 50 mmol·l^–1^ the velocities plateau (Figure 1B) because charges play an important role in the motion mechanism, as represented in Figure 1C,D. When more DMS molecules react, the rate at which charged species are generated is high. This higher rate provides a greater driving force for the propulsion of the particles; however, it also raises the ionic strength at the interface of the particle with the solution (Figure S7B). As a result, the electrical double layer of the particles, known as Debye length, becomes saturated and compressed. A compressed Debye length allows the particles to come into closer contact with the glass surface due to decreased repulsion caused by ionic screening. This closer interaction slows down the particle movement, which is measured by the decrease in average velocity. At high concentrations, this process dominates and ions accumulate in solution, which means that during the length of the video the velocities of particles will gradually decrease, resulting in a decline of average velocities (Figure 1A). At all concentrations, the motion is Cu-cap forward.

**Figure 1:**
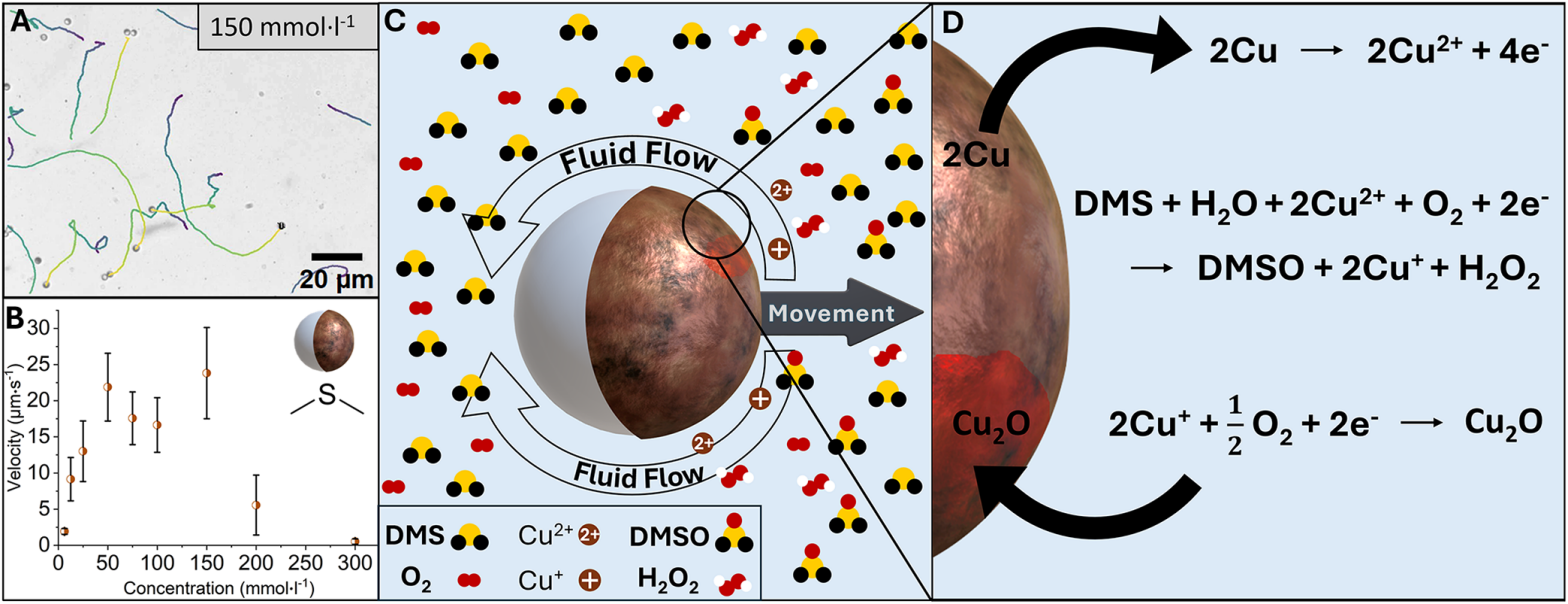
Trajectories of Cu@SiO_2_ Janus particles in 150 mmol·l^–1^ of DMS tracked over 17 s (A) with the averaged velocities of particles over a wide range of concentrations (B). A proposed reaction scheme of the Cu@SiO_2_ in suspension facilitating the oxidation of DMS into DMSO and producing H_2_O_2_ (C) with detailed suggested reaction mechanisms simultaneously happening in the liquid and on the solid particle (D).

Figure 1D shows one possible proposed reaction mechanism (see Figure S4 for the step-by-step reactions): Copper metal is known to readily oxidize its surface in humid environments, and the Cu-cap of the Cu@SiO_2_ Janus particle interacts with the aqueous solution in the same way leading to a partial formation of Cu^2+^ ions in solution. These ions coordinate to DMS through the sulfur atom, forming a transient complex that facilitates the oxidation of DMS by dissolved oxygen to yield DMSO. NMR analysis of the reaction liquid confirmed the presence of a mixture of DMS and DMSO with singlet peaks at 2.06 and 2.68 ppm, respectively (Figure 2C). During this reaction, the 2Cu^2+^ ions are reduced into 2Cu^+^ ions, which are comparatively less stable in aqueous solutions. These charged Cu ions play an important role in the propulsion of the microswimmer as the Cu^2+^ diffuses from the Cu-cap and subsequently through the reaction mechanism get consumed. This process creates a local gradient of charged species that helps to create osmotic flows to actuate the particle. This occurs in conjunction with the diffusion of the largest reaction product—DMSO, which diffuses away from the Cu-cap of the Janus particle, creating a chemical gradient. Chemical gradients around the particle also generate osmotic flows, which propel the particle due to its asymmetric nature. Concurrently, dissolved oxygen is also reduced to H_2_O_2_ via reacting with H_2_O_2_. Although H_2_O_2_ is a well-studied chemical fuel for micromotors, here it is produced at much lower concentrations and would only have a small synergistic effect on top of the sulfide propulsion (even at the highest concentration of DMS, the maximal achieve-able concentration of H_2_O_2_ would be <0.35%(*v*/*v*), which is below typical values for fuel concentrations^32^). The remaining 2 Cu^+^ ions form a Cu_2_O precipitate upon their reaction with oxygen (Figure 2A). We assume these tends to nucleate on the metallic copper surface to form solid deposits because the formed insoluble Cu_2_O deposits were confirmed by X-ray diffractometry, analyzing pre- and postreaction copper surfaces Figure 2A. Metallic copper shows peaks at 43.4, 50.5, and 74.2°, while the postreaction sample showed additional peaks at 29.7, 36.6, 42.5, 61.5, 73.6, and 74.2°, indicative of a metallic copper and cuprite mixture. The formed Cu_2_O is suggested to also acts as a catalyst^33^ for the breakdown of the H_2_O_2_ byproduct, forming H_2_O and O_2_ (Figure 4). Precipitation of Cu^+^ ions helps to limit the ionic content that the reaction produces, allowing the propulsion to occur for longer before reaching a high ionic state for the particles to get adsorbed onto the surface of the substrate, similar to a halogen-copper redox mechanism described by Henze et al.^34^ The overall reaction generates chemical gradients of DMS/DMSO and the Cu^2+^ ions in a thin layer around the reactive Cu-cap of the particle, driving osmotic flows that propel the particle forward. Despite the precipitation of Cu_2_O, the reaction produces more ions than it consumes, likely due to the reactivity of H_2_O_2_, and this increases the ionic strength in the bulk solution over time as the ions diffuse away from the reaction site. Consequently, the solution’s conductivity increases (Figure S7B), while the pH remains fairly stable. The slight downward pH trend is seen in all measured conditions and can be attributed to the dissolution of CO_2_ from air, as even the control samples with no DMS show a comparable decrease (Figure S7A). Note that alternative reaction routes such as the direct generation of copper­(I) ions, followed by their disproportionation or stabilization by sulfur-containing compounds, can also be ascribed to the NMR results. This process would involve the creation of peroxide ions, which could in the following oxidize DMS. The analytical results presented could also indicate these alternative pathways, and the evidence presented is insufficient to conclusively distinguish between these alternatives.
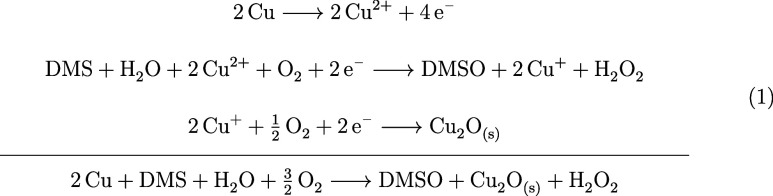


**Figure 2:**
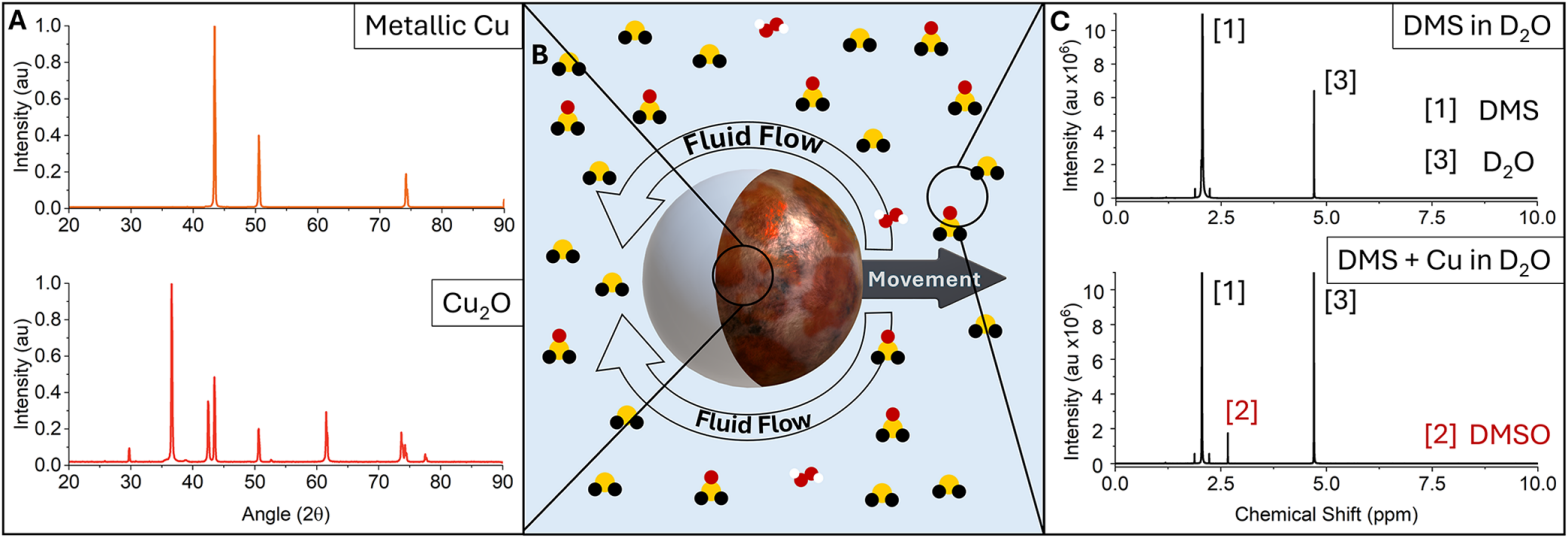
Suggested scheme of a Cu@SiO_2_ Janus particle being propelled by a catalytic reaction using DMS in solution, with the reactive copper hemisphere facing forward (B), with the reaction products reflected by the XRD and NMR analyses in panels (A) and (C), respectively.

To verify the conversion of the DMS fuel to DMSO, a colorimetric assay adopted from Grigsby and Palamand^35^ was used to selectively complex a nitroprusside ion with DMS to form a pink/purple colored complex solution with a maximal absorption at 536 nm (Figure S6). When an aqueous solution of DMS was incubated with copper powder, the detected DMS concentration postreaction in the assay was reduced proportionally to the mass of copper used and the time of incubation. The limit of detection of the DMS-nitroprusside complex was found to be 1.5 mmol·l^–1^. When the concentration of DMS exceeded 12.5 mmol·L^–1^, DMS reacted with the copper powder according to the proposed mechanism (eq 1 and the step-by-step outline in Figure S4). This reaction led to the formation of a passivation layer of less reactive Cu_2_O on the copper surface, thereby reducing the rate of oxidation of Cu_(s)_ to Cu^2+^. Consequently, the oxidation of DMS to DMSO was limited, resulting in a nonlinear relationship between the added DMS concentration and the absorption of the complex. Lastly, the concentration of the sodium nitroprusside was chosen to ensure the DMS would not fully saturate the nitroprusside ions in solution, which would plateau the absorption beyond a certain concentration of DMS.

When modifying the body properties (particle size and thickness of Cu-cap), we find that particles with a thicker cap show slightly higher velocity values, especially at higher fuel concentrations, until they are trapped at the substrate at 300 mmol·l^–1^ (see Figure S5 for 10 and 50 nm Cu-caps).

Additionally, with the velocity data of 3 μm SiO_2_ particles with a 30 nm Cu-cap, we investigated the swimming behavior of 2 and 5 μm particles with the same cap thickness. The 5 μm particles show the slowest velocities, followed by 3 μm and finally the 2 μm, in agreement with the relation v ∝1/*R* stated by Ebbens et al. and other groups^36–38^ (Figure S5 for size of Janus particles vs velocity plots). This is an interesting result, as it means that although the reaction mechanism in Ebbens et al. was catalytic and the one demonstrated here is a redox reaction involving the cap material, they are both limited by the diffusion rate of reactants to the reactive surface of the particle.

DMS is one of the most abundant sulfides in whisky, but other homologues also contribute to the overall taste and smell. To investigate the effects of chemically modifying the fuel structure, we selected other sulfides with varying carbon chain lengths and tested their ability to cause propulsion (Figure 3). Note that longer side chains are associated with lower solubility in water, e.g., DMS ≈ 350 mmol·l^–1^ ^39^ whereas dipropyl sulfide (DPS) ≈ 3 mmol·l^–1^.^40^ A full list of all tested compounds is available in Table S1. Diethyl sulfide (DES) (Figure 3A,D) reaches the second highest velocities at 17 mmol·l^–1^, slowing as it approaches its maximal solubility limit. Although its maximal velocity is slightly lower than that of DMS, it shows a similar pattern of velocities against concentration. Because its diffusion is slower due to its molecular size compared to DMS, the chemical gradient that it forms is steeper, explaining why it can propel the microswimmers to similar velocities as DMS at a 10× lower concentration. The strong dependence on concentration indicates that surface reaction kinetics are not the rate-limiting process. DPS has a maximal solubility at the STP of only 3 mmol·l^–1^, which limits the velocity of particles in suspension. Both DMS and DES show low velocities (∼3 μm·s^–1^) at this concentration. The low solubility of DPS in water in conjunction with the slowest rate of reaction with copper due to its size explain why the velocities stay very low across the concentration range, see Figure 3F. Despite having the slowest diffusion of all tested sulfides, the concentration gradient generated by DPS can still actuate the microswimmers, albeit at slow velocities (compared to DMS’s diffusion coefficient at 20 ^o^C being 1.19 × 10^–9^ m^2^·s^–1^ ^41^ ). To further understand the balance of diffusion coefficients, reactivity, and solubility in the propulsion of sulfide-driven active matter, Methyl Propyl Sulfide (MPS) was used. This molecule combines an intermediate solubility (33 mmol·l^–1^) with one longer aliphatic carbon chain than the other, and has a very similar molar mass to DES (Figure 3B,E). Its velocity profile against concentration follows a similar pattern of DPS rather than those of DMS or DES. This suggests the reactivity near the surface of the microswimmer is potentially the limiting factor in this reaction, causing the velocities to increase only slightly when higher concentrations of fuel were used. This could be explained by the structure of the molecule, the shorter methyl side chain donates less electron density to the sulfur, making it more electrophilic and thus more reactive compared to the longer propyl side chains of DPS. As hypothesized, the higher reactivity of MPS allows it to actuate our microswimmers to greater velocities of about ∼7.5 μm·s^–1^, when compared to DPS (∼3 μm·s^–1^). Nonetheless, the reaction rate is still likely the limiting factor indicated by the velocity profile against concentration. NMR measurements of the solutions postreaction with copper were taken, all of which showed an oxidized version of the respective fuel (Table S2) and Figure S9. This confirms a reaction mechanism analogous to that of DMS.

**Figure 3:**
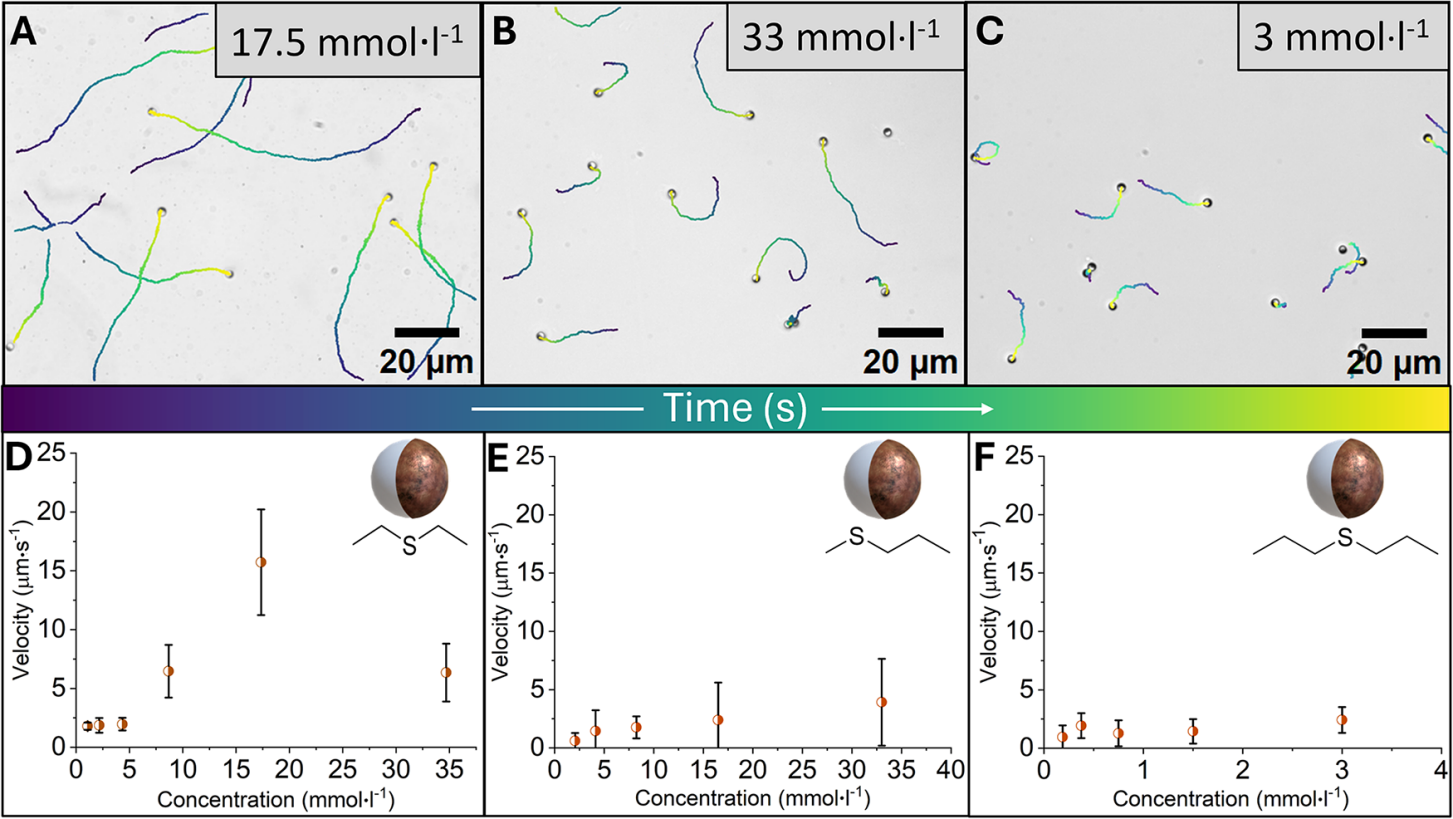
Trackplots of DES (A), MPS (B), and DPS (C) showing the trajectory of each particle in their respective fuel solution over 17 s. Averaged velocities of 3 μm Cu@SiO_2_ Janus particles in a range of concentrations of DES (D), MPS (E), and DPS (F).

To test the limitations of this swimming mechanism, other sulfur-containing molecules were investigated for their capacity to propel Cu@SiO_2_ Janus particles. In order to distinguish between slow swimming speeds and Brownian motion, single particle video tracking analysis was used to evaluate mean square displacements (MSD, using a custom python code, see Experimental Section). If the particles underwent Brownian motion, the first eq (eq 2) would apply, resulting in a straight line of the plotted MSD versus lag time. However, if the particles moved with directed active motion, the second equation applies, and from the power of second term in the equation, it can be seen why it forms a ballistic curve. Only DMS, DES, DPS, and MPS showed ballistic curves (α > 1) of MSD vs lag time (Figure 4A), indicative of active motion. The remaining tested fuels showed only diffusive or subdiffusive motion (Figure 4B). These parameters allow us to characterize the type of motion each fuel induces on the Cu@SiO_2_ Janus particles and differentiate actively moving particles from diffusive ones.

2 $$\begin{array}{l} r^{2}=4Dt^{\alpha }\\ r^{2}=4\mathrm{Dt}+{\left(\mathrm{Vt}\right)}^{2} \end{array}$$

where *r* is the mean squared displacement (MSD), *D* is the diffusion coefficient, *t* is time, α is the anomalous diffusion exponent, and *V* is the drift velocity.^42–44^

**Figure 4:**
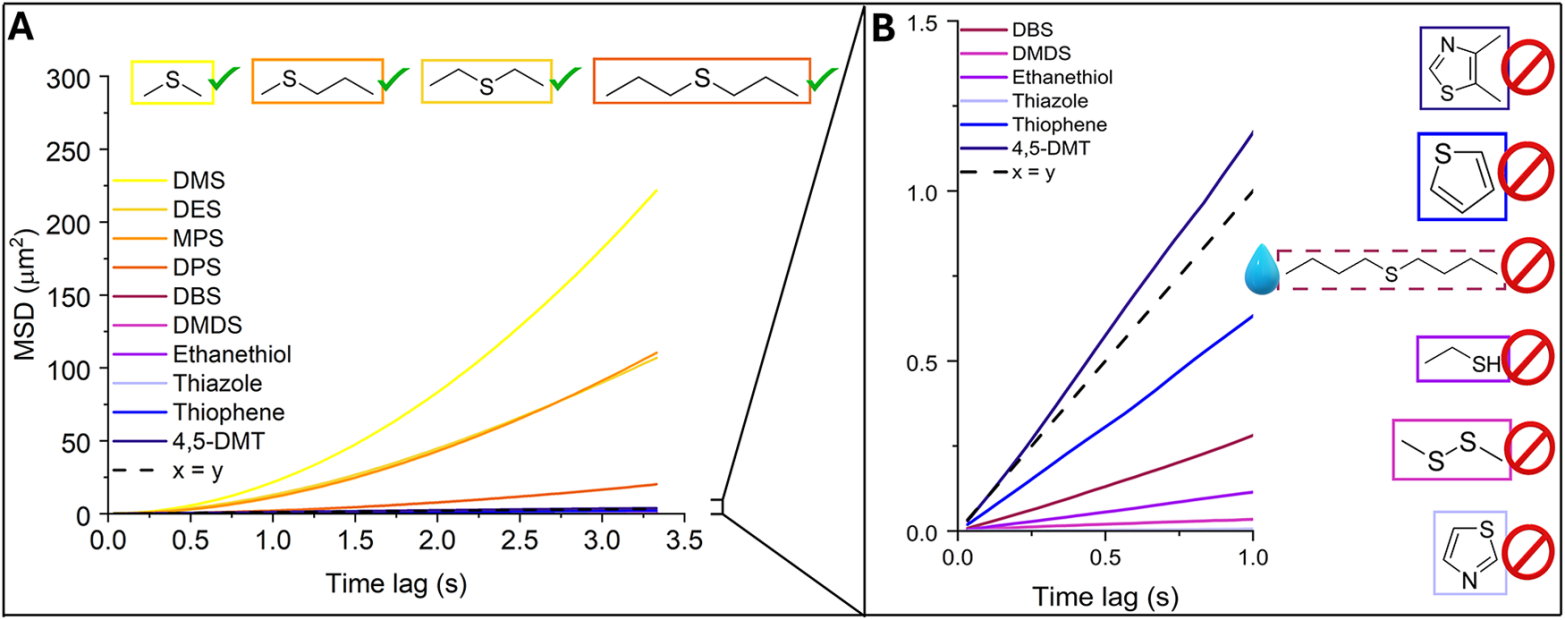
Summarized MSD plots of all tested sulfur-containing molecules and their effect on the propulsion of Cu@SiO_2_ Janus particles. While DMS, DES, DPS and MPS (A) have a ballistic curve demonstrating active motion, the chemicals on the right (B) have a linear curve geometry, demonstrating a Brownian or sub-Brownian type of motion.

The other categories of chemicals containing sulfur in whisky (Figure 4B), e.g., thiazole, thiophene, ethanethiol, and dimethyl disulfide, were tested as well. All of these showed linear MSD vs lag time curves, a hallmark of Brownian motion. Only 4,5-Dimethyl Thiazole (4,5-DMT) showed a similar slope to α = 1, whereas the rest of them showed an α < 1, subdiffusive motion regime. It is worth noting that Dibutyl Sulfide (DBS) despite its structural similarity to other aliphatic sulfides fails to effectively propel motion of copper Janus particles. We hypothesize that this is due to the very low solubility of this aliphatic sulfide in water at the STP, 0.3 mmol·l^–1^, so that no sufficient chemical gradient can be generated.

Sulfide fuels effectively propel Janus particles in aqueous solutions. Inspired by whisky, we further investigated their behavior in an ethanol–water mixture as the swimming medium (Figure S8). The emergence of Marangoni flows formed by the local surface tension differences promises interesting effects. These convective flows also have the potential to carry yet unsedimented particles and drag them in the flow, which poses a significant difficulty for the particles’ velocity video analysis. Focusing on sedimented particles, close to the substrate (less affected by Marangoni flows), adding even a small amount of ethanol into the experiment containing Cu@SiO_2_ and fuel suspension quickly stalls the movement of particles. Additionally, when the sulfide fuel solution contains >50% (v/v) ethanol, we observe that the particles get stuck to the glass substrate. There are several factors that cause this to occur, the dielectric constant of the medium is reduced (at 25 °C, Ethanol = 24.3, Water = 78.54) shrinking the screening length (Debye length) of the particle’s and substrate’s electric double layers, which increases the likelihood of their EDLs overlapping and aggregating. A lower dielectric constant in the medium also causes a discharge of ions from the EDLs, reducing the surface charge magnitude, which makes the suspension less stable due to reduced electrostatic repulsion forces. Additionally, the viscosity of water–ethanol mixtures has been widely reported to be greater than that of pure ethanol or water, reaching up to ≈ 2.147× the viscosity of pure water. Although micrometer-sized Janus particles are assumed to be force- and torque-free and hence viscosity should not influence propulsion, viscosity dependent effects are known for several microswimmers and cannot be completely excluded here. The reactivity of the micromotors appears to be preserved at least to some degree even when we do not observe their motion. We deduce this from the 90° orientation in which they are stuck (Figure S8), showing that the particles react with the fuel solution but almost instantaneously adhere on the surface. Another possible contribution to this phenomenon comes from long-range hydrophobic attraction, which was enhanced by the presence of ethanol. Because of the above reasons, it is impossible to test Cu@SiO_2_ Janus particles in more realistic or even real whisky samples, regardless of them containing the chemical fuels we have demonstrated to actuate Cu@SiO_2_ Janus particles in aqueous solutions. We have shown that sulfides, in addition to being central compounds for whisky properties, have the ability to function as fuel for copper micromotors. These belong to the newer class of redox micromotors, similar to our previously published halogen-copper redox chemistry-driven Cu@SiO_2_ Janus particles.^34^ Compared with conventional catalytic Janus microswimmers that rely on H_2_O_2_ or H_2_N_2_ ^26,32,38^ for propulsion, aliphatic sulfides offer a distinct advantage in that they are substantially less hazardous and less corrosive, despite their characteristic odor at the used concentrations. They also achieve greater actuation velocities with fuel concentrations on the mmol·l^–1^ scale. The material combination of our active matter, Cu and SiO_2_, is less expensive than bimetallic Janus particles or ones using more precious metals.^23,25,26^ Moreover, aliphatic sulfides offer tunability in their chain lengths that translates into different velocity profiles. The drawbacks of our system are that while the metal on the surface of microswimmers in catalytic reactions offers a surface for a reaction to occur, in this case, the Cu-cap is a part of the reagents, which over time reacts to form Cu_2_O. As our propulsion mechanism relies on a series of reactions, this can make the system more sensitive to changes in the solution that could affect the propulsion behavior. Finally, while our reactions rely on dissolved oxygen in the swimming solution, these reactions would likely not work in low-oxygen or anoxic environments.

## Conclusions

We have broadened the available fuel options for the propulsion of artificial microswimmers in aqueous environments to a memorable whisky-inspired new type of active matter. To do so, we took inspiration from an industrial process, namely, copper and sulfide reactions during whisky distillation and aging, and scaled it down to a microscopic scale. This resulted in actuation of Cu@SiO_2_ Janus particles in aqueous solutions of DMS, DES, MPS, and DPS up to 30 μm·s^–1^. We used XRD and NMR analyses to investigate the products of the proposed reaction mechanism behind the actuation of the particles. This showed an oxidation of DMS to DMSO and the formation of insoluble Cu_2_O (cuprite) deposits on the copper cap of Cu@SiO_2_ Janus particles. While other sulfur-containing molecules, e.g., thiophene, thiazole, etc., which are also present in whisky, did not demonstrate any capacity for propulsion. Within the tested sulfides, only aliphatic monosulfides were capable of serving as a chemical fuel for the Cu@SiO_2_ Janus particles in aqueous solutions. The ethanol–water mixtures used as a swimming medium showed the system’s limitations due to the colloidal nature of our system, which stalled the particle motion when ethanol was used as a solvent for the sulfide fuel. This study demonstrates how chemical reactions on an industrial scale can serve as an inspiration for expanding the types of active matter, while demonstrating that a redox reaction can be a driving force for spherical Cu@SiO_2_ Janus particles.

## Experimental Section

### Preparation of Cu@SiO_2_ Janus Microspheres

The Janus particles were prepared using 2, 3, and 5 μm solid SiO_2_ spheres purchased from Sigma-Aldrich. 50 μL particle suspension was drop-casted onto an oxygen plasma cleaned 24 × 24 mm glass slide and dried in air at ambient temperature. Once fully dried, the coated glass slide was inserted into a physical vapor deposition machine to deposit a 30 nm layer of thermally discharged copper. Coated slides were cut into shards using a diamond-tipped pen and sonicated in water to remove adhered Janus particles from the glass slide into a water suspension. These suspensions were used as a source of Janus particles for microscopy experiments.

### Optical Microscopy Setup

A Zeiss microscope Axio 5 with a DFK camera and an Axio Imager 2 with a Thor Cam camera were used with diascopic illumination of the samples. Suspensions of microsized Janus particles were placed on glass slides with PVC spacers adhered to the glass to form circular wells. And the motion of the particle was recorded at 30 fps for 500 frames.

### Scanning Electron Microscopy

SEM images of the Janus particle samples were taken using a ZEISS Gemini SEM 300 instrument with the SE detector. Samples were prepared by drop-casting 1 μL suspension in water on aluminum tape-coated sample holders. After complete drying of the water, the sample was sputtered with a thin film of Gold Palladium 80:20 to increase the conductivity.

### Velocity Tracking

Videos were recorded using a digital camera mounted on the microscope. Frames-per-second (fps) and scale of the videos allowed the movement of Janus particles in the videos to be calculated into their velocities in μm/s. This was calculated using a custom Python-coded script written by Daniel Gordon (University of Strathclyde). Additional analysis of the ballistic nature of motion was performed using the mean square displacement (MSD). The MSD is calculated from the squared distance each particle traveled over the course of the video and provides an insight into the type of motion the particle is undergoing based on the shape of the resulting curve.^42–44^ The equation that describes the relationship of MSD (*r* ^2^), diffusion (*D*), time lag (*t*), velocity (*V*), and the exponent α (eq 2) shows that if a tracked particle is purely diffusive (Brownian motion) α = 1, for constrained/subdiffusive motion α < 1 and for superdiffusive α > 1.

### Nitroprusside Colorimetric Analysis

An adjusted method used by Grigsby and Palamand^35^ was used for the colorimetric determination of DMS concentration in aqueous samples. A reagent mixture was prepared by using 1 mL of 5% (v/v) solution of sodium nitroprusside, 9 mL of water, and 1 mL of 10% NaOH. This mixture was incubated at RT for 30 min before adding a 6 M solution of HCl to acidify, which changed the pale pink solution to yellow in the absence of DMS. For DMS-containing samples, a fraction of the water was substituted for a DMS sample, which would change the final color to a bright pink/purple. This color change was measured on a UV/vis spectrometer at a wavelength of 536 nm. A calibration curve was established with known concentrations of DMS followed by unknown samples.

### NMR Method

All samples were prepared in pure D_2_O (Sigma, 1,51,882), using copper powder (Aldrich, 26,607–8) as a mimic of the Cu-cap on the Cu@SiO_2_ Janus particle with each sulfide fuel and incubated at RT for 24 h before submitting for NMR analysis. ^1^H NMR spectra were taken using a Bruker AV400 Nano instrument at 400 MHz with a Bruker 5 mm *SmartProbe* ^*TM*^ instrument at 300 K. A single pulse sequence was used with an acquisition time of 2.04 s, a 30° flip angle, and a relaxation delay of 1 s. Sixteen scans were taken after two dummy scans with a spectral width of 8012 Hz.

### XRD Method

XRD patterns were acquired using a Bruker 2D phaser in a 2θ range of 20–90°, with symmetrical scans. The copper powder samples were dispersed in ethanol and drop-cast onto a silicon wafer. The copper sample incubated with an aqueous solution of DMS for an hour changed color to a darker brown from the typical reddish-brown color of pure copper. The copper sample in pure water did not show any changes of color.

## Supplementary Material











## Notes

Additional figures (SEM images, preparation schemes, NMR spectra, experimental details), and a supporting information table of chemicals used and their NMR peaks (PDF)
Video 1: 3 μm Janus particles in 6.25 to 300 mmol·l^–1^ of DMS (MP4)
Video 2: 3 μm Janus particles in 17 mmol·l^–1^ of DES (AVI)
Video 3: 3 μm Janus particles in 33 mmol·l^–1^ of MPS (AVI)
Video 4: 3 μm Janus particles in 3 mmol·l^–1^ of DPS (AVI)
