# Supplementary material for: Whisky-Inspired Active Matter
Source: ACS Appl Mater Interfaces. 2026 Apr 20;18(18):26998–7005. doi: 10.1021/acsami.6c00489 (PMC13181724; doi:10.1021/acsami.6c00489)
Supplement: Supplementary file 1 [file am6c00489_si_001.pdf]

Supporting Information
Whisky-inspired Active Matter

Khalifa Mohamed,[†] Kelly Henze,[‡] and Juliane Simmchen[*,†,‡]

†*Department of Pure and Applied Chemistry, University of Strathclyde, Glasgow, G1 1XL, United Kingdom*

‡*Department of Chemistry, TU Dresden, Dresden, 01069, Germany*

E-mail: juliane.simmchen@strath.ac.uk

Supporting Videos

All videos were recorded at 30 fps using a digital microscope-mounted camera.

- SI Video 1: 3 μm Janus particles in 6.25 to 300 $mmol \cdot l^{-1}$ of DMS
- SI Video 2: 3 μm Janus particles in 17 $mmol \cdot l^{-1}$ of DES
- SI Video 3: 3 μm Janus particles in 33 $mmol \cdot l^{-1}$ of MPS
- SI Video 4: 3 μm Janus particles in 3 $mmol \cdot l^{-1}$ of DPS

Supporting Files

- Figure S1: Scottish whisky-making process overview
- Figure S2: SEM images of pre- and post-reaction $Cu@SiO_2$ Janus particles
- Figure S3: $Cu@SiO_2$ Janus particle preparation scheme
- Table S1: Chemical Information

- Figure S4: Detailed step-by-step equation of DMS with Cu
- Figure S5: Varying size and Cu thickness of Cu@SiO_2 Janus particles
- Figure S6: Colorimetric DMS determination using sodium nitroprusside
- Figure S7: pH and conductivity measurements
- Figure S8: Ethanol addition to active matter experiments
- Table S2: ^{1}H NMR spectra peaks of all reactions causing propulsion
- Figure S9: Full ^{1}H NMR spectra of sulfide fuels propelling Janus microswimmers

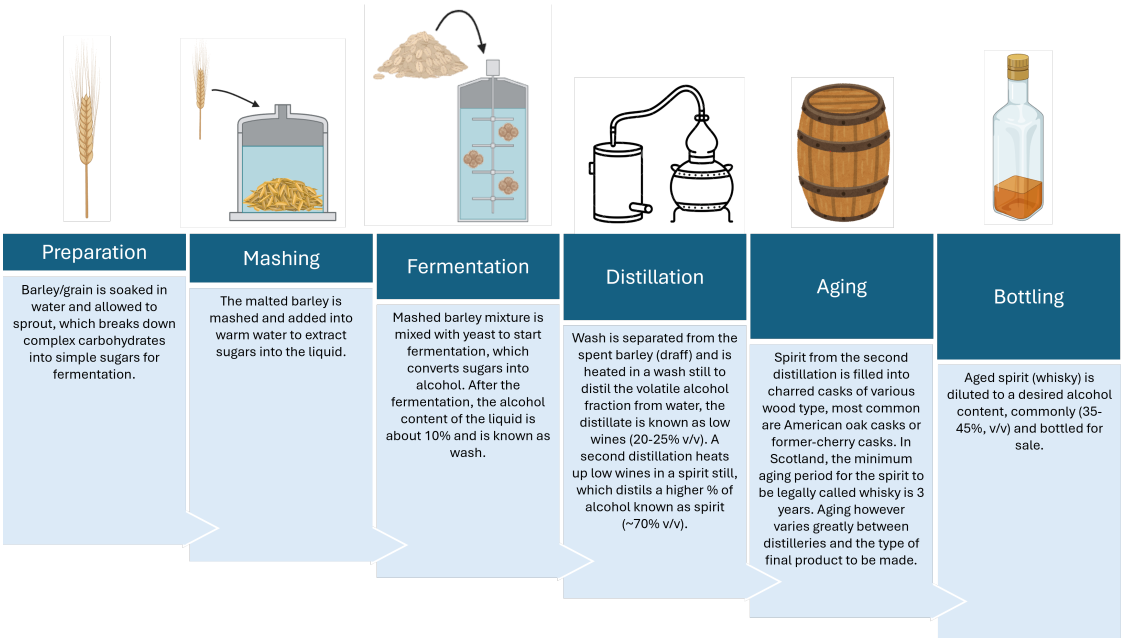

Figure S1: A simplified overview of a Scottish whisky-making process, including some of the specific Scottish terms that might be unfamiliar to a wider population. Information was sourced from several manufacturers of Scottish whisky.[1–3] This figure was created using BioRender.com and Canva.com

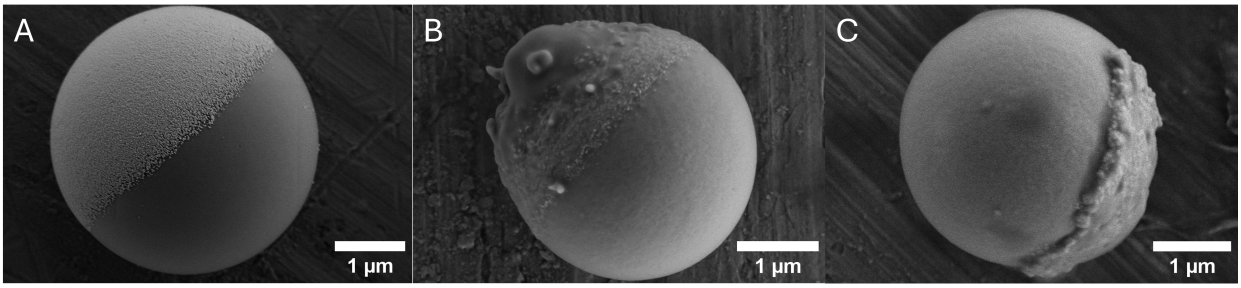

Figure S2: SEM images of 3μm Cu@$\mathrm{SiO_2}$ Janus particles pre- (A) and post-reaction with 150 $mmol \cdot l^{-1}$ DMS and 17 $mmol \cdot l^{-1}$ DES

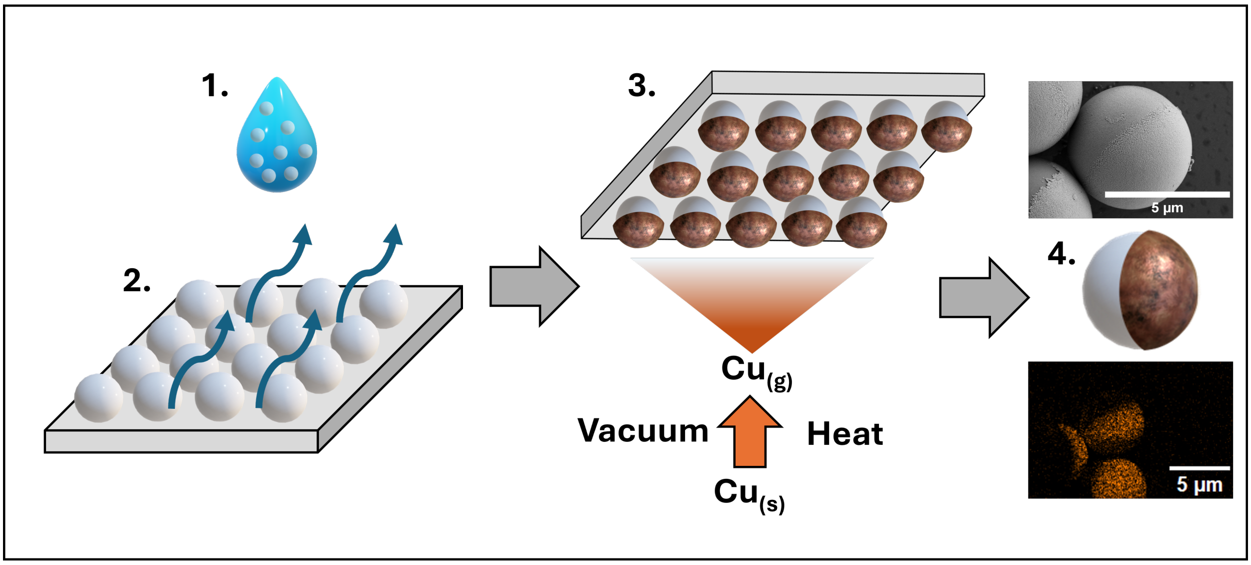

Figure S3: Preparation method of Cu@$\mathrm{SiO_2}$ Janus particles. First step is prepare a water suspension of spherical $\mathrm{SiO_2}$ particles of a desired size, e.g. 3μm. The suspension is drop-casted onto a glass slide at a concentration to form a monolayer of particles. The liquid is left to evaporate, and a vapor deposition technique is used to evaporate copper powder using vacuum and heat, which covers the surface of the particle-covered slide above the evaporation crucible. The thickness of the deposited copper can be controlled to create various Cu-cap thicknesses. Copper-coated particles are sonicated from the glass slide to release monodisperse Cu@$\mathrm{SiO_2}$ Janus particles into a suspension. SEM and elemental analysis show the localization of the copper on one hemisphere of the final particle.

$$2\,\mathrm{Cu} \longrightarrow 2Cu^{2+} + 4\,\mathrm{e^-}$$

$$\mathrm{DMS} + 2\,\mathrm{Cu^{2+}} \xrightleftharpoons{\text{weak}} 2\,\mathrm{Cu^{2+}[DMS]}$$

$$2\,\mathrm{Cu^{2+}[DMS]} + \mathrm{H_2O} + \mathrm{O_2} + 2\,\mathrm{e^-} \longrightarrow \mathrm{DMSO} + 2\,\mathrm{Cu^+} + \mathrm{H2O2}$$

$$2\,\mathrm{Cu^+} + \tfrac{1}{2}\,\mathrm{O_2} + 2\,\mathrm{e^-} \longrightarrow \mathrm{Cu_2O_{(s)}}$$

$$\text{potential synergistic contribution: } \mathrm{H_2O_2} \xrightarrow{\mathrm{Cu_2O}} \mathrm{H_2O} + \tfrac{1}{2}\,\mathrm{O_2}$$

Figure S4: A detailed reaction scheme showing all of the proposed subsequent steps of DMS reacting with Cu from the Cu-cap of a Cu@$\mathrm{SiO_2}$ Janus particle. Chemical species in gray signify reaction intermediates that are consumed in the reaction by a subsequent step.

Table S1: Summary table of all sulfur-containing molecules that were tested for reactivity with Cu@SiO$_2$ Janus particles

Abbreviation	Chemical Name	CAS Number	Supplier	Product Code
DMS	Dimethyl Sulfide	75-18-3	Merck	471577
DES	Diethyl Sulfide	352-93-2	Merck	107247
MPS	Methyl Propyl Sulfide	3877-15-4	Apollo Scientific	OR1029206
DPS	Dipropyl Sulfide	111-47-7	Apollo Scientific	OR0793
DBS	Dibutyl Sulfide	544-40-1	Merck	51491
DMDS	Dimethyl Disulfide	624-92-0	Merck	471569
Thiazole	Thiazole	288-47-1	Merck	151645
4,5-DMT	4,5-Dimethyl Thiazole	3581-91-7	Merck	W327409
Thiophene	Thiphene	110-02-1	Merck	T31801
Ethanethiol	Ethanethiol	75-08-1	Fisher Scientific	10306940

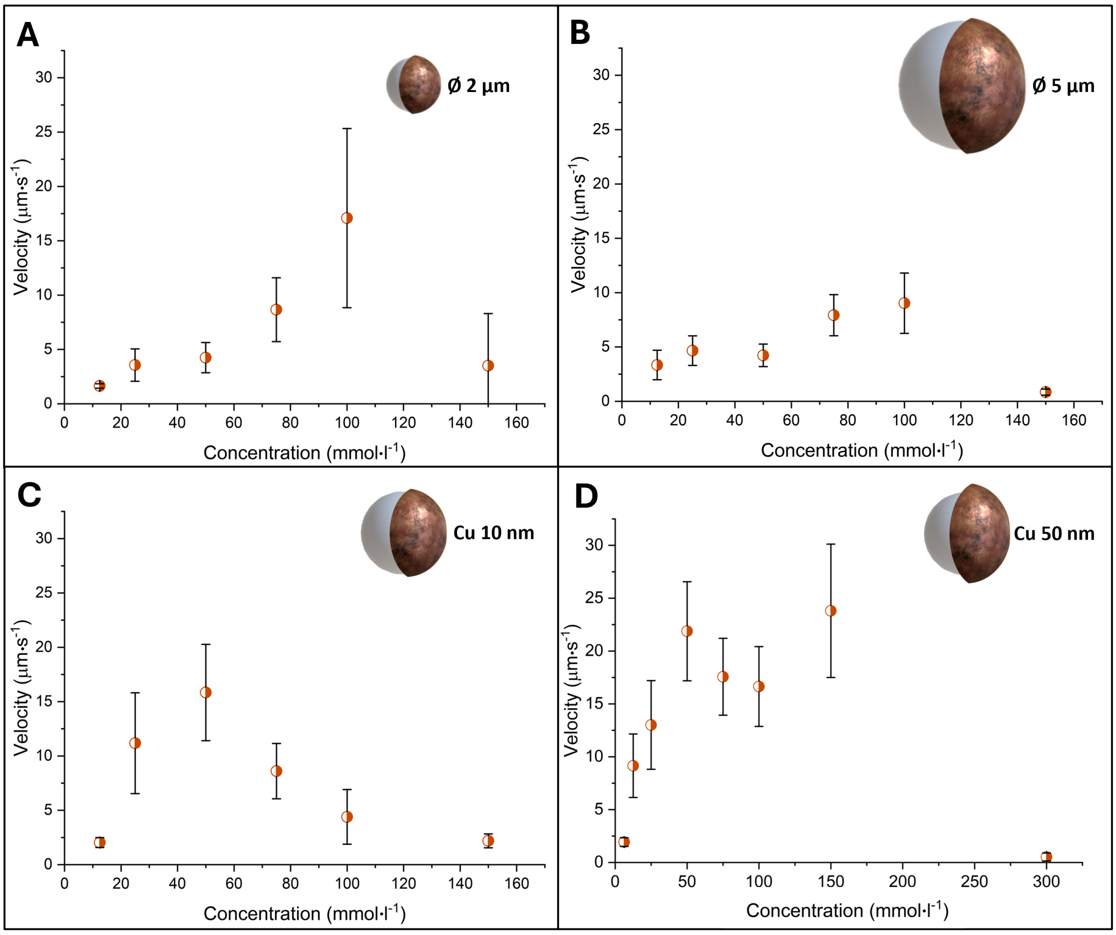

Figure S5: Velocity plots of spherical Cu@SiO$_2$ Janus particles of diameters 2 (A) and 5 μm (B) with a 30 nm layer of copper metal deposited and 3 μm diamater particle with 10 (C) and 50 nm (D) layers of copper in a range of aqueous DMS solution concentrations

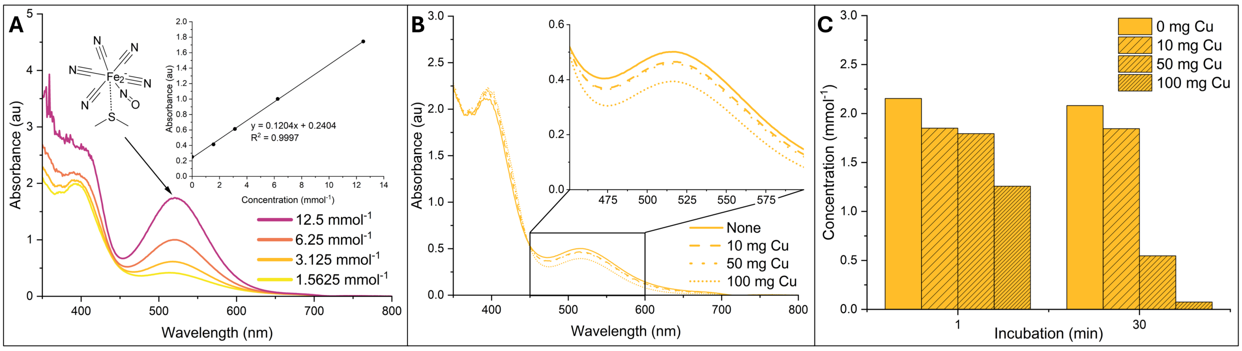

Figure S6: A colourimetric assay on the basis of the nitroprusside ion complexation to DMS, yielding a pink/purple complex with an absorbance at 536 nm, adapted from the work of Grigsby and Palamand.[4] A concentration curve between 1.5 and 12.5 $mmol \cdot l^{-1}$ of DMS showed a linear dependency of absorbance on the concentration with an $R^2 = 0.9997$. (A) 3.125 mM of DMS was incubated with different masses of Cu powder (B), Calculated concentrations of DMS when incubated with different masses of Cu powder after 1 and 30 min, using the linear curve equation from panel A (C).

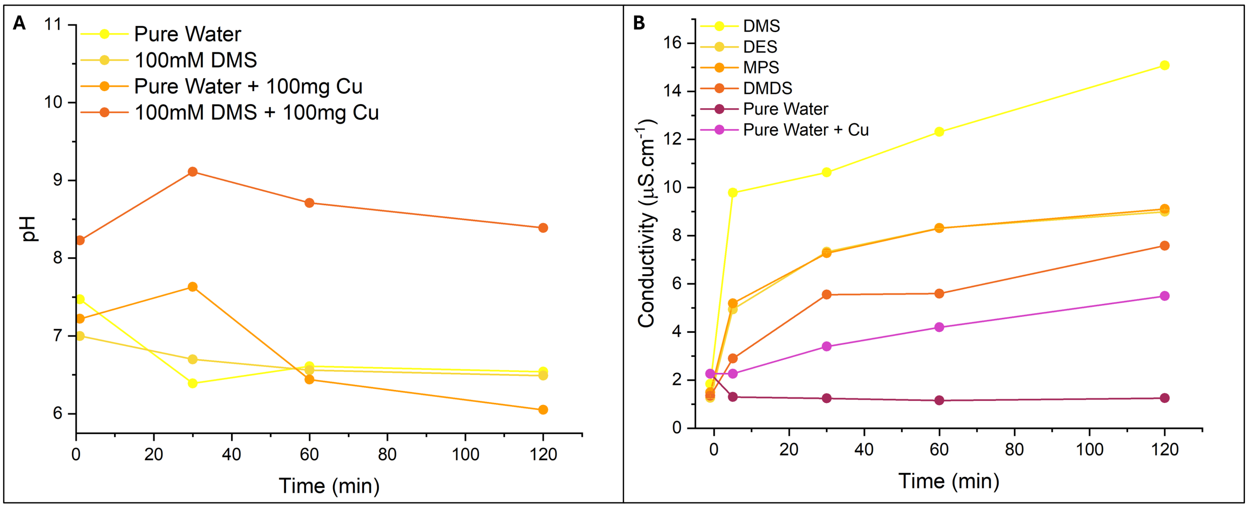

Figure S7: Changes of pH (A) and conductivity (B) of solutions over time using different sulfides when incubated with Cu. Pure water/pure water with Cu powder act as controls.

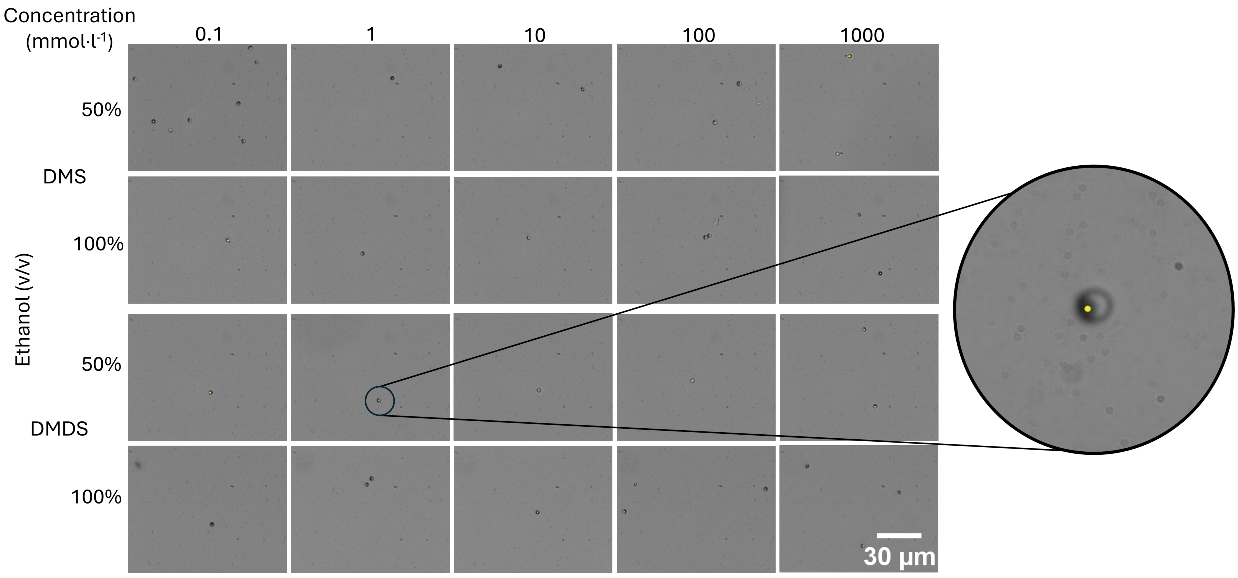

Figure S8: Trackplots of 3μm in diameter Cu@SiO_2 Janus particles in different concentrations of DMS and DMDS with 50 and 100% Ethanol demonstrating that no active motion is observable because particles get immediately stuck on the substrate, most likely because no effective double layer can form.

Table S2: NMR results (^1H, D_2O, 400 MHz) of fuel solutions before and after reaction with copper in D_2O

Reaction	Peak Type (Chemical shift (ppm))	Identified Compounds
DMS only	Singlet (2.68)	DMS
DMS+Cu	Singlet (2.06), Singlet (2.68)	DMS and DMSO
DES only	Triplet(1.17), Quartet(2.54)	DES
DES+Cu	Triplet(1.17), Triplet(1.24), Quartet(2.79), Quartet(2.85)	DES and DESO
DPS only	Triplet(0.9), Sextet(1.55), Triplet(2.38), Triplet(2.47)	DPS
DPS+Cu	Triplet(0.9), Triplet(1.00), Sextet(1.55), Triplet(2.5), Triplet(2.79)	DPS and DPSO
MPS only	Triplet (1.00), Triplet(1.17), Triplet (1.33), Singlet(2.05), Quartet(2.36), Quartet(2.46), Quartet(2.53), Quartet(2.71)	
MPS+Cu	Triplet(0.74), Triplet(0.9), Triplet(1.0), Sextet(1.55), Sextet(1.7), Singlet(1.86), Singlet(2.04), Singlet(2.21), Singlet(2.39),Triplet(2.47), Singlet(2.63), Triplet(2.81)	MPS and MPSO*

* Possible trace amounts of MES and MESO

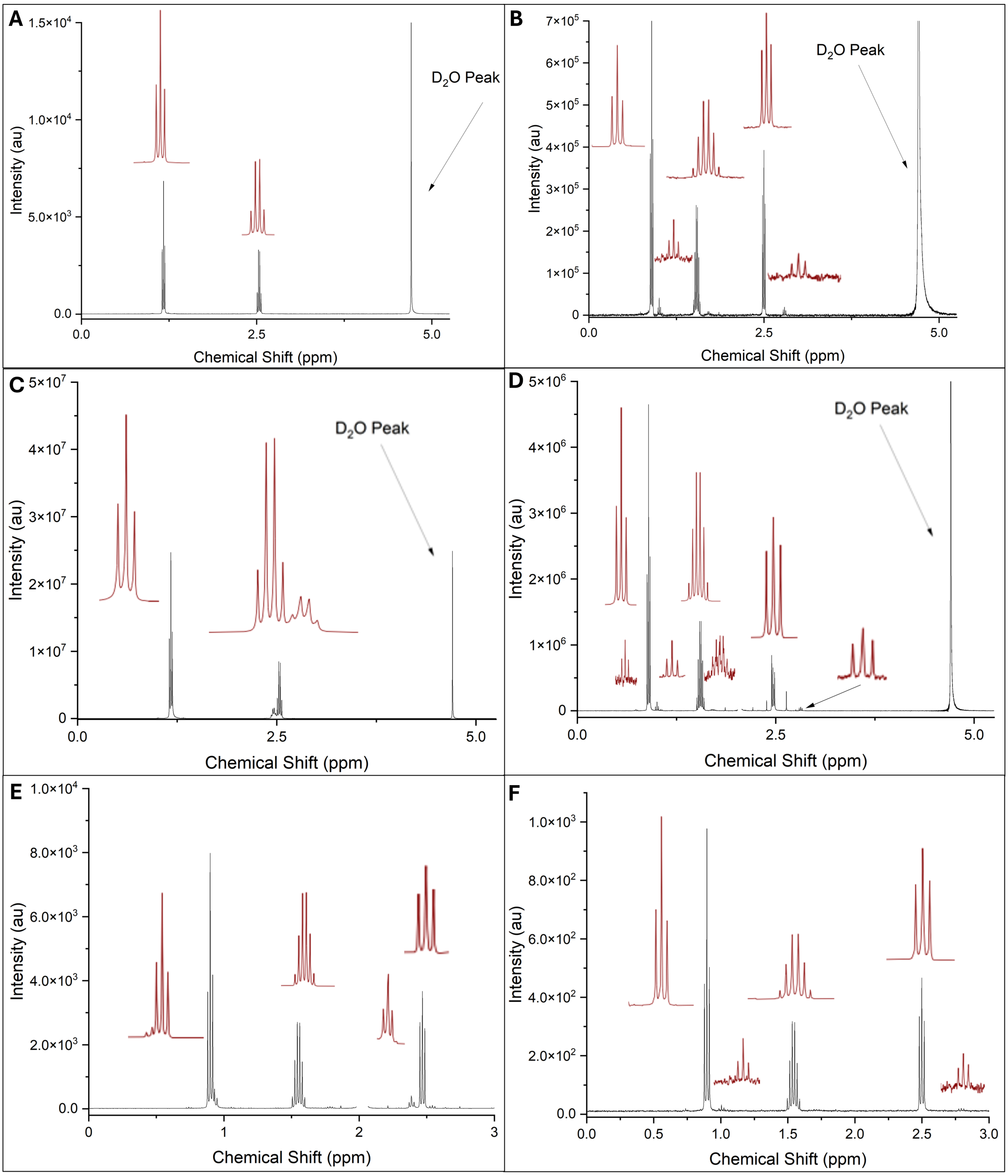

Figure S9: Full NMR spectra of the additional sulfide fuels that successfully showed propulsion of Cu@SiO_2 Janus particles. The cutouts above each peak detail its peak structure.

References

(1) Laphroaig Distillery How to Make Whisky. https://www.laphroaig.com/how-to-make-whisky, 2026; Accessed: 2026-01-08.

(2) Scotch Whisky industry records £5.4bn global exports in 2024 amid 'turbulent' global trading conditions. https://www.scotch-whisky.org.uk/newsroom/2024-export-figures/.

(3) The Scotch Whisky Experience Whisky Making. https://www.scotchwhiskyexperience.co.uk/about/about-whisky/whisky-making/, 2026; Accessed: 2026-01-08.

(4) Grigsby, J.; Palamand, S. A colorimetric procedure for the measurement of dimethyl sulfide in water, wort, and beer. *Journal of the American Society of Brewing Chemists* **1977**, *35*, 43–48.
